# 488. Initial laboratory markers may help distinguish between Kawasaki Disease and Multisystem Inflammatory Syndrome in Children

**DOI:** 10.1093/ofid/ofad500.557

**Published:** 2023-11-27

**Authors:** Shir Miretzky, Katharine N Clouser, Sejal M Bhavsar, Aryeh Z Baer, Anna Carmela Sagcal-Gironella, Jamie M Pinto, Meghan Tozzi, Vaidehi Patel

**Affiliations:** Hackensack University Medical Center, New York, New York; Hackensack University Medical Center, Hackensack, New Jersey; Hackensack University Medical Center, Hackensack, New Jersey; Hackensack University Medical Center, New York, New York; Hackensack University Medical Center, New York, New York; K. Hovnanian Children's Hospital at Jersey Shore University Medical, Neptune, New Jersey; Hackensack University Medical Center, New York, New York; The Herman and Walter Samuelson Children's Hospital at Sinai, Baltimore, Maryland

## Abstract

**Background:**

In May of 2020, the Centers for Disease Control and Prevention defined a new diagnosis, Multisystem Inflammatory Syndrome in Children (MIS-C), described as a Kawasaki-like illness and related to the SARS-CoV-2 virus. Patients with this syndrome met criteria for inflammation, along with multiple organ system involvement. Clinically distinguishing between Kawasaki Disease (KD) and MIS-C is challenging due to significant overlap between the two disease entities. Furthermore, approximately 25-50% of MIS-C cases also meet criteria for KD. Currently there is very limited data describing specific differences in laboratory markers between KD and MIS-C to guide clinicians in diagnosis and management. This study aims to assess if quantitative laboratory data markers help distinguish between KD and MIS-C.

**Methods:**

We conducted a retrospective study of patients hospitalized within two network pediatric hospitals with the diagnosis of MIS-C [M35.81] or Kawasaki Disease [M30.3] between January 1, 2019 and March 31, 2021. Children with an alternate diagnosis at discharge were excluded. Demographic characteristics and laboratory data were recorded and analyzed.

**Results:**

A total of 91 pediatric patients were included in the study: 40 with MIS-C and 45 with KD. Patients with MIS-C were significantly older with a median age 5.3 years, compared to 2.4 years for KD (*p*=0.001). Patients with KD presented with higher median white blood cell (WBC) count, absolute lymphocyte count, and platelet count compared to those with MIS-C (p< 0.001). A higher alanine aminotransferase (ALT) was also noted (p=0.01). Patients with MIS-C were noted to have higher median ferritin levels and C-reactive protein (CRP) (p< 0.001) (Table 1).

**Figure:**
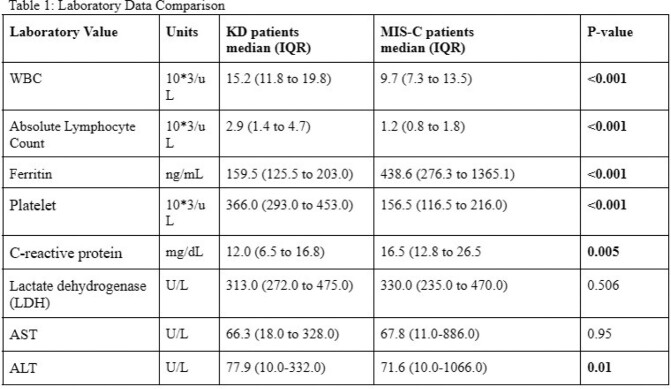

**Conclusion:**

Both MIS-C and KD are autoinflammatory disease processes that have significant overlap. Identifying differences in initial laboratory values upon presentation can lead to earlier diagnosis. A normal ferritin level may indicate KD.

**Disclosures:**

**All Authors**: No reported disclosures

